# Post‐operative recovery of quality‐of‐life following percutaneous nephrolithotomy: The impact on pain intensity and interference and the ability to participate in social roles

**DOI:** 10.1002/bco2.70102

**Published:** 2025-11-29

**Authors:** Justin B. Ziemba, Amanda Jones, Hanna Stambakio, Gregory E. Tasian, Jing Huang

**Affiliations:** ^1^ Division of Urology, Department of Surgery, Hospital of the University of Pennsylvania, Perelman School of Medicine University of Pennsylvania Philadelphia PA USA; ^2^ Division of Urology, Department of Surgery Children's Hospital of Philadelphia Philadelphia PA USA; ^3^ Department of Biostatistics, Epidemiology, and Informatics, Perelman School of Medicine University of Pennsylvania Philadelphia PA USA

**Keywords:** Percutaneous Patient Outcome Assessment, Quality of Life Kidney Calculi Nephrolithotomy

## Abstract

**Objectives:**

To prospectively capture patient‐reported outcomes in the post‐operative period to better understand the quality‐of‐life impact of percutaneous nephrolithotomy (PNL).

**Subjects and Methods:**

Adults undergoing PNL for renal/ureteral stones were eligible for inclusion (11/2020–8/2022). Patients prospectively completed PROMIS – Pain Intensity, Pain Interference and Ability to participate in social roles and activities in‐person pre‐operatively (POD 0) and via email on POD 1, 7, 14 and 30. Scores are reported as T‐scores (normalized to US pop., mean = 50) with a change of 5 (0.5 SD) considered clinically significant.

**Results:**

A total of 62 participants enrolled at POD 0 (POD 1 = 28, POD 7 = 29, POD 14 = 20, POD 30 = 23). There was a worsening of quality of life from POD 0 to POD 1 for pain intensity (median difference 7.9, p = 0.005) and pain interference (median difference 11.9, p = 0.0003), but not for the ability to participate in social roles (median difference −5.3, p = 0.07). This was also observed for POD 0 to POD 7, but only for the dimension of pain interference (median difference 7.2, p = 0.02). All three dimensions then improved until POD 30. In multivariable analysis, there were no variables associated with severe symptomatology for any of the three dimensions.

**Conclusions:**

Pain intensity and interference sharply increase immediately post‐operatively, reducing quality of life, although the ability to participate in social roles is not impacted. The absolute magnitude of this change is significant, with a final improvement at 30 days at or above baseline. Results offer meaningful insight to assist in setting expectations for patients post‐operatively.

**Abbreviations**
PRO: Patient‐Reported Outcomes
HRQOL: Health‐Related Quality of Life
PNL: Percutaneous Nephrolithotomy
PROMIS: Patient‐Reported Outcomes Measurement Information System
SD: Standard Deviation
MCID: Minimal Clinically Important Difference
WISQOL: Wisconsin Stone Quality of Life

## 1 INTRODUCTION

Patient‐reported outcomes (PRO) are increasingly emphasized in clinical research and routine medical practice, serving to evaluate symptoms, guide treatment choices, enhance communication and monitor patient progress.^1^ A crucial PRO is health‐related quality of life (HRQOL), a concept that examines how disease and treatment potentially influence an individual's psychological, physiological and social functioning.^2^ Evaluating HRQOL is especially significant for patients with nephrolithiasis, given the variable disease progression observed over time, which may not directly align with traditional objective measures like stone‐free status on diagnostic imaging.^3^ Tracking HRQOL changes within individuals following treatment provides valuable insights for patient counselling, clinical decision‐making and outcome assessment.

Acknowledging the significance of HRQOL in nephrolithiasis patients, there is growing attention on assessing how surgical interventions impact HRQOL dimensions during post‐operative recovery. While ureteroscopy has an expanding research base demonstrating its effects on post‐operative HRQOL across dimensions such as pain severity, pain‐related functional limitations and urinary symptoms,^4^, ^5^, ^6^, ^7^, ^8^ comparable comprehensive data remain largely absent for percutaneous nephrolithotomy (PNL).^9^

To address this area of unmet need, we prospectively collected longitudinal HRQOL data on patients with nephrolithiasis following treatment with PNL, which represents a common surgical modality for the removal of large‐volume kidney stones.^10^ We specifically focused on the dimensions of pain intensity, pain interference and ability to participate in social roles to characterize recovery patterns and identify sociodemographic and surgical factors that may be associated with the risk of severe symptomatology. We hypothesize that PNL would result in a worsening in HRQOL across all 3 measured dimensions postoperatively, but these would rapidly normalize by the 7‐day measurement interval.

## 2 MATERIALS AND METHODS

### 2.1 Study Population

Similar to a prior investigation in a ureteroscopy cohort,^8^ this prospective observational study was conducted between November 2020 and August 2022. The study's participant selection criteria included adult individuals aged 18 years or older who were diagnosed with renal or proximal ureteral stones through computed tomography imaging and subsequently underwent PNL. Participants who had a nephrostomy tube or ureteral stent placed as preparatory measures for definitive stone removal were considered eligible for enrolment. The details of the surgical technique used for PNL can be found in the **Supplementary Methods,** with all participants scheduled for a 23‐hour admission and discharged with a nephrostomy tube. This nephrostomy tube was removed on post‐operative day (POD) #4–6, with a date selected depending on staff availability in the clinic. All patients were offered a 7‐day supply of the non‐narcotic pain medication meloxicam (7.5 mg daily) at discharge. Individuals were excluded from the study if they belonged to vulnerable demographic categories (including pregnant individuals, incarcerated persons, those lacking decision‐making capacity, etc.), were unable to communicate in English, lacked a functional email address and/or could not independently operate a computer or tablet device for survey administration.

### 2.2 Survey Instruments

The Patient‐Reported Outcomes Measurement Information System (PROMIS) contains a list of generic survey instruments that are available to assess numerous PRO dimensions (https://www.healthmeasures.net/). The PROMIS instruments of pain intensity (v1.0, 3a), pain interference (v1.1, computer‐adaptive test) and ability to participate in social roles and activities (v2.0, short‐form 8a) were each selected as prior research has shown the responsiveness of these instruments in patients with nephrolithiasis.^5^ In brief, pain intensity measures concepts such as pain at its worst level, average pain and current pain level with a 7‐day recall; pain interference measures concepts such as pain interfering with the enjoyment of life or close personal relationships with a 7‐day recall; and ability to participate in social roles measures concepts such as trouble doing all usual work or limitations on regular family activities with no recall period (see Supplementary Figure 1 for individual instruments and items).

### 2.3 Enrolment and Data Collection

Treatment modality for the identified stone was selected in a shared decision‐making fashion using guideline‐based criteria.^10^, ^11^ No patient underwent a planned or unplanned second‐stage procedure during the study period.

At enrolment, coinciding with the day of surgery (day 0), participants electronically completed a demographic questionnaire and all three PROMIS instruments. Subsequently, these identical PROMIS instruments were electronically re‐administered to each participant at predetermined intervals: 1, 7, 14 and 30 days following the initial enrolment, with automatic email notifications facilitating the data collection process. A reminder phone call was performed on POD #3 to confirm receipt of the POD #1 automatic email and encourage participation with the subsequent survey invitations. For consistency, all participants completed the survey electronically and independently at each post‐operative time point, even if they remained hospitalized during that period. The entirety of the study's data was gathered and organized utilizing REDCap's electronic data capture infrastructure, leveraging the Shared Data Instrument Library.^12^, ^13^, ^14^

### 2.4 Statistical Analysis

#### 2.4.1 Scoring

All scores for the PROMIS measures are reported as T‐scores. T‐scores are transformed and normalized raw scores relative to a reference United States (US) population. A T‐score of 50 represents the US population mean, with scores of 40 and 60 representing one standard deviation (SD) below and above that population, respectively. A higher score indicates more of the concept being measured. For example, for the dimension of pain interference, a score of 60 means more pain interference as compared to the US population mean score of 50. However, for social roles, a score of 60 means a greater ability to participate socially as compared to the US population mean score of 50, meaning for this outcome measure, a higher score is more favourable.

A minimal clinically important difference (MCID) was also identified/denoted for the PROMIS measures. An MCID is the smallest change in a patient‐reported outcome measure that is meaningful or relevant to the participant.^15^ In this study, we selected a conservative estimate of 0.5 SD, or 5 points, to indicate an MCID.

#### 2.4.2 Primary Analysis

The primary outcome was a MCID (5 points) change in total T‐score from enrolment (day 0) to each additional time point (day 1, 7, 14 and 30) for each PROMIS instrument. The study population was assumed to have a loss to follow‐up by a factor of 40% by day 1, 60% by day 14 and 80% by day 30 based on a prior similar study.^7^ The significance level (α‐two‐sided) of 0.05 and power of 0.8 were used to calculate a sample size. A sample size of 175 participants was required to detect a MCID of 5 points with 80% power and at a 5% significance level. Median scores on each PROMIS instrument were compared to the population norm (T‐score 50) at all time points using a Wilcoxon rank‐sum test. This non‐parametric test was chosen because it does not assume normality and is suitable for comparing medians in skewed or non‐normally distributed data, which was expected based on preliminary data review. Differences in median scores across time on each PROMIS instrument were compared using the Kruskal‐Wallis test, a non‐parametric alternative to ANOVA, which allows for comparison across multiple time points without assuming equal variances or normal distribution. For each PROMIS instrument, pairwise comparisons of median difference for each time point combination were assessed with the Wilcoxon rank‐sum test with correction for multiple comparisons.

#### 2.4.3 Secondary Analysis

A secondary multivariable analysis was performed to identify demographic and surgical factors associated with severe symptoms for each dimension, which was defined as a change of 10 points in the total T‐score from enrolment (day 0) to day 1. The complete analysis plan is available in the **Supplementary Methods**. This study followed the STROBE Statement for the reporting of observational studies.^16^ The Institutional Review Board approved this study with informed consent obtained from each participant.

## 3 RESULTS

### 3.1 Patient Demographics

A total of 62 participants were enrolled and completed a baseline PROMIS instrument assessment at day zero. The median age was 59 years (IQR 51–66). Overall, 52% (32/62) and 48% (29/62) of enrolled participants were male and female, respectively; 83% (49/62) were white; and 42% (26/62) were first‐time stone formers, with 92% (57/62) presenting with renal stones. Further baseline demographic and stone characteristics of our population are outlined in Table 1. Table 2 contains information on surgical technique. At the end of each procedure, the collecting system was visually negative on flexible nephroscopy. However, post‐operative imaging was not performed until after the 30‐day study period and thus was not included in this analysis. The median length of stay in the hospital was 1 day. A total of 10 complications (16%) occurred during the study period, which are further detailed in Table 2. A total of 100% (62/62), 45% (28/62), 47% (29/62), 32% (20/62) and 37% (23/62) of participants completed the survey instruments at 0‐, 1‐, 7‐, 14‐ and 30‐day intervals, respectively.

**TABLE 1:** Summary of Baseline Demographics and Stone Characteristics.

	(N = 62)
**Age**	
Median [IQR]	59.20 [50.57, 65.77]
**Ethnicity**	
Hispanic/Latino	1(1.639%)
Non‐Hispanic/Latino	58(95.08%)
Unknown	2(3.279%)
**Race**	
White	49(83.05%)
Black or African American	8(13.56%)
Asian	1(1.695%)
Native Hawaiian or Pacific Islander	1(1.695%)
**Gender**	
Male	32(52.46%)
Female	29(47.54%)
**BMI**	
Median [IQR]	29.37 [26.61, 34.28]
**Is this your first kidney stone**	
Yes	26(41.94%)
No	36(58.06%)
**Insurance Status**	
Commercial	31(50.00%)
Medicare	19(30.65%)
Medicaid	11(17.74%)
Federal/Military/Veterans Administration	1(1.613%)
**Laterality**	
Unilateral	62(100.00%)
**Primary/Dominant Stone Location**	
Kidney	57(91.94%)
Upper Ureter	5(8.065%)
**Primary/Dominant Stone Size (mm)**	
Median [IQR]	25.00 [20.00, 30.00]

* Branching stones were classified for both location and size based on the location of their largest or primary component.

**TABLE 2:** Summary of Operative Technique.

**Pre‐Operative Ipsilateral Ureteral Stent (N = 62)**	
Yes	7(11.29%)
No	55(88.71%)
**Post‐Operative Ipsilateral Ureteral Stent**	
Yes	8(12.90%)
No	54(87.10%)
**Pre‐Operative Ipsilateral Nephrostomy Tube**	
No	46(74.19%)
Yes	15(24.19%)
**Post‐Operative Ipsilateral Nephrostomy Tube**	
No	6(9.677%)
Yes	56(90.32%)
**Initial Access Puncture Site**	
Upper Pole	11(17.74%)
Mid Pole	15(24.19%)
Lower Pole	35(56.45%)
**Total Operative Time (min) [Scope In to Scope Out]**	
Median [IQR]	121.00 [97.50, 139.75]
**Complications**	
Clavien‐Dindo 1	3 (Self‐Limited Hematuria)
Clavien‐Dindo 2	3 (Urinary Tract Infection) 1 (Transient Ischemic Attack)
Clavien‐Dindo 3	2 (Pseudoaneurysm) 1 (Colonic Perforation)
Clavien‐Dindo 4	0
Clavien‐Dindo 5	0
Total	10 (16.13%)

### 3.2 Longitudinal Comparisons

Table 3 reports the scores for each PRO at each time point. There was a significant change in pain intensity and pain interference, but not in the ability to participate in social roles. At baseline, as compared to the normalized population median score, there was a statistically and clinically (MCID) significant decrease in pain intensity, but no difference was observed for either pain interference or the ability to participate in social roles. At the conclusion of the study period (day 30), as compared to the normalized population median score, there was a statistically and clinically significant decrease in pain intensity and an increase in the ability to participate in social roles (i.e. both better than the normal reference population), but no difference was observed for pain interference.

**TABLE 3:** Summary of Scores by Dimension by Time.

	Day 0	Day 1	Day 7	Day 14	Day 30	*p‐value
	N = 62	N = 28	N = 29	N = 20	N = 23	
**Pain Intensity T‐Score**						
Median	40.5	48.4	47.8	42.6	40.5	0.02
[Min, Max]	[30.7, 57.5]	[30.7, 60.6]	[30.7, 66.0]	[30.7, 52.3]	[30.7, 60.6]	
^compared to the population median score (50)	<0.001	0.76	0.05	0.01	0.004	
**Pain Interference T‐score**						
Median	50.1	62.1	57.3	38.7	55.4	<0.001
[Min, Max]	[38.7, 71.6]	[38.7, 76.4]	[38.7, 76.4]	[38.7, 66.9]	[38.7, 69.6]	
^compared to the population median (50)	0.74	0.001	0.008	0.48	0.42	
**Ability to Participate in Social T‐Score**						
Median	51.7	46.5	48.9	56.6	56.6	0.10
[Min, Max]	[25.9, 65.4]	[25.9, 65.4]	[36.9, 65.4]	[25.9, 65.4]	[37.6, 65.4]	
^compared to the population median (50)	0.06	0.21	0.80	0.07	0.04	

* Differences in median across time are compared using the Kruskal–Wallis test.

^P‐values of differences between the data and population median score (50) using the Wilcoxon test.

**FIGURE 1:**
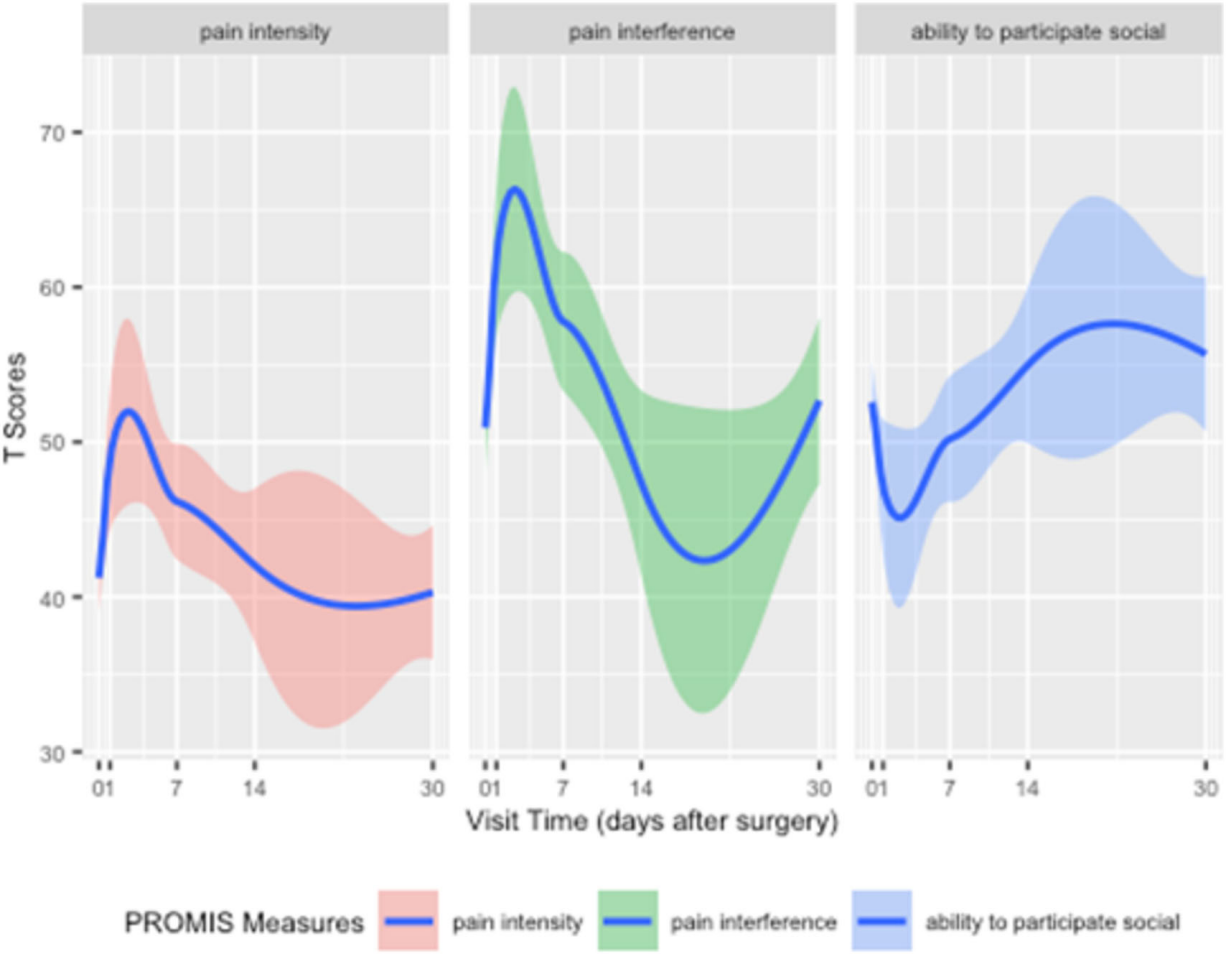
Smooth plot of scores for each dimension over the study period. Smoothing curves fitted using the LOESS method, with a solid line representing the median score and a shaded area representing the 95% confidence interval.

In pairwise comparisons from enrolment (POD #0), there was a statistically and clinically significant worsening quality of life from POD #0 to POD #1 for pain intensity (median difference 7.9, p = 0.005) and pain interference (median difference 11.9, p = 0.0003), but not for the ability to participate in social roles (median difference −5.3, p = 0.07). A similar trend with a statistically and clinically significant worsening quality of life was observed from POD #0 to POD #7, but only for the dimension of pain interference (median difference 7.2, p = 0.02). All three dimensions transitioned from a decline to an improvement in quality of life after POD #7 with a continued increase in scores in pairwise comparisons from POD #0 to POD #14 and POD #0 to POD #30, although these were not statistically significant. Figure 1 depicts smooth curves to visualize the change in scores for each dimension across the study period. All pairwise comparisons at each time point combination and for each dimension are available in Supplementary Table 1.

### 3.3 Univariate and Multivariable Analysis

The associations of demographic and surgical characteristics on multivariable analysis for each instrument at each time point are available in **Supplementary Table** 2. **Supplementary Table** 3 shows the covariates in the final multivariable model for severe symptoms (delta T‐score >10) at post‐operative day 1. In the final reduced multivariable model, there were no variables associated with severe symptomatology for the dimensions of pain intensity, pain interference or ability to participate in social roles.

## 4 DISCUSSION

In this cohort of participants undergoing PNL, we found that pain intensity is statistically and clinically significantly better at baseline, but no different from a reference US adult in either pain interference or ability to participate in social roles. This may explain why pain intensity on post‐operative day 1 is worse than at baseline, but still about equal to the US mean, whereas pain interference is a full standard deviation above a reference US adult. This recovery pattern of pain intensity and pain interference is consistent with observations in a ureteroscopy cohort.^7^ Interestingly, by post‐operative day 30, both pain intensity and the ability to participate in social roles are statistically and clinically significantly better compared to a reference US adult, but no different for pain interference. We also observed that HRQOL worsens from enrolment to POD #1, which continues until at least post‐operative day 7. However, after POD #7 there is a transition from worsening to a continual and sustained improvement in HRQOL until the study end at POD #30. The absolute magnitude of this change during this 30‐day period is profound, with each dimension ultimately improving over one standard deviation from POD #1. Assessment of demographic, stone and surgical characteristics revealed no characteristic was associated with severe symptoms at POD #1; however, this may be due to the limited sample size, which is underpowered to detect such differences.

While there are numerous cross‐sectional studies examining HRQOL in nephrolithiasis,^7^, ^17^, ^18^, ^19^, ^20^, ^21^, ^22^ very few have focused exclusively on the changes to HRQOL during the post‐operative recovery after surgical interventions.^4^, ^5^, ^7^, ^23^, ^24^, ^25^ Even fewer measure HRQOL longitudinally following surgical treatment using PROMIS,^5^, ^6^, ^7^, ^8^ and none have focused on PNL. In fact, the available studies examining HRQOL after PNL have focused almost exclusively on the technical aspects of the operation, such as mini vs standard tract size or post‐operative nephrostomy tube vs ureteral stent placement.^26^, ^27^, ^28^

In the only study that focuses exclusively on assessing HRQOL following PNL, the SF‐36 questionnaire was selected and administered at 2 weeks pre‐operatively, and then at 3 months and 1 year post‐operatively.^29^ At baseline, all of the eight dimensions and the physical and mental health component summary scores were decreased.^29^ However, by 3 months post‐operatively, the dimensions of physical function, role physical, bodily pain, mental health and the physical component summary score each experienced statistically significant improvement.^29^ This trend continued for these same dimensions, but with the addition of social functioning at 1 year post‐operatively.^29^ Interestingly, the only dimension that achieved a statistically significant and clinically important improvement at both 3 months and 1 year was bodily pain, although role physical and physical component summary score improved significantly at 1‐year.^29^ No analysis was performed to determine if there were associated demographic, stone or procedural characteristics that may have influenced these findings.

Unlike this prior study, in our cohort, we observed that pre‐operative HRQOL was preserved (no different) for all dimensions. This difference can potentially be attributed to the metrics selected between the two studies. The SF‐36 is a generic global health survey that assesses several broad categories, while the dimensions assessed in this study are generic, yet were selected to be responsive to changes in HRQOL as a result of kidney stone surgery.^5^, ^7^ In addition, it is certainly plausible that the underlying impact of large‐volume kidney stones on HRQOL may have evolved in the decade between the prior and current study, where patients are identified incidentally rather than due to symptomatic presentation, which is seen in other urological disease states.

Although our study focuses on the more immediate post‐operative period and utilizes different HRQOL measures, there are similarities, particularly an improvement in post‐operative pain indices in both cohorts. In the historical cohort, the only dimension that achieved a statistically and clinically significant improvement at both 3 months and 1 year was bodily pain.^29^ In our cohort, we similarly observed that the largest absolute magnitude of change in pairwise comparisons was POD #1 to POD #14 for pain interference and for POD #1 to POD #30 for pain intensity. It is not surprising that an improvement in HRQOL concepts focused on pain would be observed after surgical intervention, particularly for larger volume stones with the removal of the stone itself, and recovery from the surgery.

This is similarly observed in other surgical modalities like ureteroscopy (URS). For example, in the study by Talwar et al., which examined a cohort undergoing URS at baseline and then repeatedly on post‐operative days POD #1, 7 and 14 with measures of pain intensity and interference, there was evidence of an immediate worsening of HRQOL at POD #1 and 7, but then a recovery by POD #14 for each measure.^7^ A similar trend was also observed for adolescents and adults undergoing ureteroscopy with ureteral stent placement who participated in the STENTS study, which assessed stent‐associated symptoms daily until stent removal through a variety of repeated instruments, including pain intensity and interference.^6^, ^25^ Pain intensity and pain interference significantly increased post‐operatively, peaked on POD #1 and 2, and then marginally decreased each subsequent day, although each dimension remained elevated until stent removal.^6^

Traditionally, only pain and bother from urinary symptoms are examined in studies evaluating the post‐operative recovery following surgical stone removal, primarily after ureteroscopy.^6^, ^7^, ^22^, ^25^ However, this represents a relatively narrowed focus on the impact of surgical therapy. We expanded this focus by including the dimension of the ability to participate in social roles and activities to provide a more holistic assessment of how surgery may impact personal, family and work relationships. No other study has examined this specific dimension in the PNL population. However, a randomized controlled study comparing post‐operative nephrostomy tube to ureteral stent drainage did rely on the use of the Wisconsin Stone Quality of Life (WISQOL) instrument, which does measure closely related concepts of social event participation, missed work or family time and less interest in socializing.^27^ In this study, they observed a significant decrease in these HRQOL dimensions in the ureteral stent group relative to the nephrostomy tube group.^27^ In addition, the nephrostomy tube group demonstrated an improvement in HRQOL across these dimensions, suggesting that drainage may play an important role in recovery.^27^ In contrast, we observed a very modest effect of PNL on recovery with respect to the ability to participate in social roles and activities, as the only pairwise comparison with significant improvement was observed from POD #1 to POD #30. This discrepancy may be attributed to the different dimensions assessed, or that our entire cohort only had post‐operative nephrostomy tube drainage, which, interestingly, did not seem to impact changes in HRQOL, as no significant difference was observed relative to the POD #7 timepoint that is directly following nephrostomy tube removal. Nevertheless, this added dimension provides important contextual information for patients to utilize in preparing for how their individual recovery may impact those around them, as they may not be able to join in on their usual activities at work, home and leisure.

No prior studies have attempted to examine the association of demographic or stone characteristics with HRQOL in the PNL population. Unfortunately, our final multivariable model did not identify any demographic or surgical characteristics associated with a risk of severe symptoms on POD #1, which may be due to a relatively small sample size, although this study was not powered to detect these differences.

We recognize several limitations. Our participant selection process excluded individuals who indicated difficulty completing the PROMIS survey instruments electronically, potentially introducing a selection bias favouring more technologically adept participants. Additionally, we experienced difficulty in recruiting the required number of participants for adequate power, despite a 2‐year recruitment window, which was at least partially due to pandemic‐related forces, and as a result, the expected and modelled participant attrition at each follow‐up interval further reduced our cohort, despite implementing electronic reminders and follow‐up calls. Despite this smaller cohort, we were able to demonstrate statistically and clinically meaningful changes in the measured dimensions, although it is certainly possible that at least some of the null findings between time points and on the final multivariable model assessing severe symptomatology may be due to this limited statistical power. We were also not able to correlate stone‐free status to our outcomes as we did not have post‐operative imaging available, as this was routinely done outside the 30‐day study window. Finally, we did not capture data regarding analgesic requirements or post‐operative urinary symptoms, which could potentially explain some variation in the measured dimensions.

We acknowledge the inherent limitation in any self‐report study, including non‐response bias, which may have skewed the results, particularly if non‐respondents' experiences significantly diverged from those who completed the surveys. In future similar studies, it would be reasonable to structure additional automated email reminders and personal outreach calls to encourage participation to increase the response rate. We acknowledge the potential confounding influence of participants' broader health status. Factors such as acute non‐urologic medical exacerbations or progression of chronic comorbid conditions could potentially contribute to observed HRQOL fluctuations. However, attempting to isolate changes exclusively related to nephrolithiasis would be impractical, as HRQOL assessment is inherently self‐reflective, holistic and complex.^2^

Despite these methodological constraints, we maintain that our findings represent a significant advancement in comprehensively characterizing patient‐reported experiences following PNL. These insights can serve as a valuable resource for clinicians in counselling and preparing patients about the potential physical, psychological and emotional outcomes associated with this surgical intervention.

## 5 CONCLUSIONS

PNL demonstrates a dramatic initial impact on HRQOL, characterized by substantial deterioration within the first 24 hours. However, this negative trajectory rapidly transforms, with a marked recovery emerging by day 7 and progressively continuing through at least day 30. The scale of this transformation is clinically meaningful, with patients ultimately experiencing HRQOL levels equivalent to or potentially exceeding their pre‐surgical baseline. Such insights provide guidance for both patients and healthcare professionals in establishing realistic expectations and understanding the nuanced recovery associated with PNL.

## AUTHOR CONTRIBUTIONS

All authors have approved the final draft and made a significant contribution to the methods and findings in the paper.

## CONFLICT OF INTEREST STATEMENT

GET is on the Scientific Advisory Board for Alnylam Pharmaceuticals and NovoNordisk Pharmaceuticals. All other authors declare no conflicts of interest.

## Supporting information


**Supplementary Figure 1:** PROMIS Instruments (derived from PDF available at https://www.healthmeasures.net/explore-measurement-systems/promis).

**Supplementary Table 1.** Pairwise comparison of median difference using Wilcoxon test.
**Supplementary Table 2.** Multivariable analysis of the association of demographic and stone characteristics on quality of life domain scores.
**Supplementary Table 3.** Final multivariable model for severe symptoms (delta T‐score >10) at post‐operative day 1.

**Data S1.** Supporting Information.
