# Supplementary material for: Post‐operative recovery of quality‐of‐life following percutaneous nephrolithotomy: The impact on pain intensity and interference and the ability to participate in social roles
Source: BJUI Compass. 2025 Nov 29;6(12):e70102. doi: 10.1002/bco2.70102 (PMC12663602; doi:10.1002/bco2.70102)
Supplement: Supplementary file 2 — Supplementary Table 1. Pairwise comparison of median difference using Wilcoxon test. Supplementary Table 2. Multivariable analysis of the association of demographic and stone characteristics on quality of life domain scores. Supplementary Table 3. Final multivariable model for severe symptoms (delta T‐score >10) at post‐operative day 1. [file BCO2-6-e70102-s001.docx]

**Supplementary Table 1. Pairwise comparison of median difference using Wilcoxon test.**

| outcome | group1 | group2 | n1 | n2 | p | median1 | median2 | median_difference |
| --- | --- | --- | --- | --- | --- | --- | --- | --- |
| pain intensity | preop_visit_arm_1 | 1_day_postop_arm_1 | 60 | 15 | 0.005000 | 40.50 | 48.40 | 7.90 |
| pain intensity | preop_visit_arm_1 | 7_day_postop_arm_1 | 60 | 23 | 0.070000 | 40.50 | 47.80 | 7.30 |
| pain intensity | preop_visit_arm_1 | 14_day_postop_arm_1 | 60 | 13 | 0.663000 | 40.50 | 42.60 | 2.10 |
| pain intensity | preop_visit_arm_1 | 30_day_postop_arm_1 | 60 | 17 | 0.709000 | 40.50 | 40.50 | 0.00 |
| pain intensity | 1_day_postop_arm_1 | 7_day_postop_arm_1 | 15 | 23 | 0.268000 | 48.40 | 47.80 | -0.60 |
| pain intensity | 1_day_postop_arm_1 | 14_day_postop_arm_1 | 15 | 13 | 0.027000 | 48.40 | 42.60 | -5.80 |
| pain intensity | 1_day_postop_arm_1 | 30_day_postop_arm_1 | 15 | 17 | 0.007000 | 48.40 | 40.50 | -7.90 |
| pain intensity | 7_day_postop_arm_1 | 14_day_postop_arm_1 | 23 | 13 | 0.305000 | 47.80 | 42.60 | -5.20 |
| pain intensity | 7_day_postop_arm_1 | 30_day_postop_arm_1 | 23 | 17 | 0.048000 | 47.80 | 40.50 | -7.30 |
| pain intensity | 14_day_postop_arm_1 | 30_day_postop_arm_1 | 13 | 17 | 0.444000 | 42.60 | 40.50 | -2.10 |
| pain interference | preop_visit_arm_1 | 1_day_postop_arm_1 | 59 | 18 | 0.000312 | 50.10 | 62.05 | 11.95 |
| pain interference | preop_visit_arm_1 | 7_day_postop_arm_1 | 59 | 23 | 0.023000 | 50.10 | 57.30 | 7.20 |
| pain interference | preop_visit_arm_1 | 14_day_postop_arm_1 | 59 | 13 | 0.287000 | 50.10 | 38.70 | -11.40 |
| pain interference | preop_visit_arm_1 | 30_day_postop_arm_1 | 59 | 16 | 0.543000 | 50.10 | 55.35 | 5.25 |
| pain interference | 1_day_postop_arm_1 | 7_day_postop_arm_1 | 18 | 23 | 0.134000 | 62.05 | 57.30 | -4.75 |
| pain interference | 1_day_postop_arm_1 | 14_day_postop_arm_1 | 18 | 13 | 0.001000 | 62.05 | 38.70 | -23.35 |
| pain interference | 1_day_postop_arm_1 | 30_day_postop_arm_1 | 18 | 16 | 0.017000 | 62.05 | 55.35 | -6.70 |
| pain interference | 7_day_postop_arm_1 | 14_day_postop_arm_1 | 23 | 13 | 0.010000 | 57.30 | 38.70 | -18.60 |
| pain interference | 7_day_postop_arm_1 | 30_day_postop_arm_1 | 23 | 16 | 0.202000 | 57.30 | 55.35 | -1.95 |
| pain interference | 14_day_postop_arm_1 | 30_day_postop_arm_1 | 13 | 16 | 0.195000 | 38.70 | 55.35 | 16.65 |
| ability to participate social | preop_visit_arm_1 | 1_day_postop_arm_1 | 61 | 22 | 0.076000 | 51.70 | 46.45 | -5.25 |
| ability to participate social | preop_visit_arm_1 | 7_day_postop_arm_1 | 61 | 26 | 0.221000 | 51.70 | 48.90 | -2.80 |
| ability to participate social | preop_visit_arm_1 | 14_day_postop_arm_1 | 61 | 17 | 0.300000 | 51.70 | 56.60 | 4.90 |
| ability to participate social | preop_visit_arm_1 | 30_day_postop_arm_1 | 61 | 17 | 0.350000 | 51.70 | 56.60 | 4.90 |
| ability to participate social | 1_day_postop_arm_1 | 7_day_postop_arm_1 | 22 | 26 | 0.525000 | 46.45 | 48.90 | 2.45 |
| ability to participate social | 1_day_postop_arm_1 | 14_day_postop_arm_1 | 22 | 17 | 0.054000 | 46.45 | 56.60 | 10.15 |
| ability to participate social | 1_day_postop_arm_1 | 30_day_postop_arm_1 | 22 | 17 | 0.045000 | 46.45 | 56.60 | 10.15 |
| ability to participate social | 7_day_postop_arm_1 | 14_day_postop_arm_1 | 26 | 17 | 0.099000 | 48.90 | 56.60 | 7.70 |
| ability to participate social | 7_day_postop_arm_1 | 30_day_postop_arm_1 | 26 | 17 | 0.103000 | 48.90 | 56.60 | 7.70 |
| ability to participate social | 14_day_postop_arm_1 | 30_day_postop_arm_1 | 17 | 17 | 0.943000 | 56.60 | 56.60 | 0.00 |

**Supplementary Table 2. Multivariable analysis of association of demographic and stone characteristics on quality of life domain scores.**

**Multivariable analysis on Day 0.**

**Pain Intensity**

| row_names | Estimate | Std. Error | t value | Pr(>\|t\|) |
| --- | --- | --- | --- | --- |
| (Intercept) | 44.618 | 6.813 | 6.549 | 0.000 |
| demo_raceBlack or African American | 7.082 | 3.340 | 2.120 | 0.039 |
| demo_raceAsian | 13.147 | 8.605 | 1.528 | 0.133 |
| demo_raceNative Hawaiian or other Pacific Islander | 18.109 | 9.368 | 1.933 | 0.059 |
| demo_kidneyYes | -5.678 | 2.263 | -2.509 | 0.015 |
| demo_age | -0.039 | 0.111 | -0.351 | 0.727 |

**Pain Interference**

| row_names | Estimate | Std. Error | t value | Pr(>\|t\|) |
| --- | --- | --- | --- | --- |
| (Intercept) | 55.515 | 7.833 | 7.087 | 0.000 |
| demo_raceBlack or African American | 9.785 | 3.805 | 2.572 | 0.013 |
| demo_raceAsian | 13.845 | 9.742 | 1.421 | 0.161 |
| demo_raceNative Hawaiian or other Pacific Islander | 17.507 | 10.673 | 1.640 | 0.107 |
| demo_kidneyYes | -7.499 | 2.594 | -2.891 | 0.006 |
| demo_age | -0.054 | 0.128 | -0.425 | 0.673 |

**Ability to participate social**

| row_names | Estimate | Std. Error | t value | Pr(>\|t\|) |
| --- | --- | --- | --- | --- |
| (Intercept) | 50.466 | 7.897 | 6.390 | 0.000 |
| demo_raceBlack or African American | -7.884 | 3.856 | -2.045 | 0.046 |
| demo_raceAsian | -12.357 | 9.885 | -1.250 | 0.217 |
| demo_raceNative Hawaiian or other Pacific Islander | -12.079 | 10.830 | -1.115 | 0.270 |
| demo_kidneyYes | 4.873 | 2.604 | 1.871 | 0.067 |
| demo_age | 0.026 | 0.129 | 0.204 | 0.839 |

**Multivariable analysis on Day 1.**

**Pain Intensity**

| row_names | Estimate | Std. Error | t value | Pr(>\|t\|) |
| --- | --- | --- | --- | --- |
| (Intercept) | 51.328 | 12.291 | 4.176 | 0.014 |
| demo_raceBlack or African American | -7.197 | 7.941 | -0.906 | 0.416 |
| demo_ethnicityUnknown | -0.293 | 9.627 | -0.030 | 0.977 |
| intra_insuranceMedicare | -5.166 | 9.147 | -0.565 | 0.602 |
| intra_insuranceMedicaid | -1.635 | 6.044 | -0.270 | 0.800 |
| intra_insuranceFederal/Military/Veterans Administration | -7.838 | 5.567 | -1.408 | 0.232 |
| intra_dominant_stone_size | 0.010 | 0.456 | 0.023 | 0.983 |
| intra_postop_stentYes | -11.534 | 5.523 | -2.088 | 0.105 |
| intra_same_day_dispoYes | 6.697 | 3.758 | 1.782 | 0.149 |

**Pain Interference**

| row_names | Estimate | Std. Error | t value | Pr(>\|t\|) |
| --- | --- | --- | --- | --- |
| (Intercept) | 40.466 | 4.256 | 9.508 | 0.000 |
| demo_ethnicityUnknown | -3.955 | 6.283 | -0.629 | 0.542 |
| demo_genderWomen | 9.962 | 2.602 | 3.829 | 0.003 |
| intra_dominant_stone_size | 0.730 | 0.177 | 4.134 | 0.002 |
| intra_same_day_dispoYes | 4.178 | 3.016 | 1.386 | 0.193 |

**Ability to participate social**

| row_names | Estimate | Std. Error | t value | Pr(>\|t\|) |
| --- | --- | --- | --- | --- |
| (Intercept) | 41.328 | 18.147 | 2.277 | 0.038 |
| demo_age | 0.143 | 0.314 | 0.456 | 0.655 |
| intra_insuranceMedicare | 16.945 | 6.731 | 2.517 | 0.024 |
| intra_insuranceMedicaid | 9.521 | 7.117 | 1.338 | 0.201 |
| intra_insuranceFederal/Military/Veterans Administration | 6.691 | 9.349 | 0.716 | 0.485 |
| intra_dominant_stone_size | -0.451 | 0.290 | -1.554 | 0.141 |

**Multivariable analysis on Day 7.**

**Pain Intensity**

| row_names | Estimate | Std. Error | t value | Pr(>\|t\|) |
| --- | --- | --- | --- | --- |
| (Intercept) | 62.077 | 10.741 | 5.780 | 0.000 |
| demo_age | -0.283 | 0.178 | -1.587 | 0.128 |
| intra_dispo_meds___9Yes | 16.175 | 8.664 | 1.867 | 0.077 |

**Pain Interference**

| row_names | Estimate | Std. Error | t value | Pr(>\|t\|) |
| --- | --- | --- | --- | --- |
| (Intercept) | 49.771 | 5.257 | 9.467 | 0.000 |
| intra_dominant_stone_size | 0.424 | 0.229 | 1.851 | 0.079 |

**Ability to participate social**

| row_names | Estimate | Std. Error | t value | Pr(>\|t\|) |
| --- | --- | --- | --- | --- |
| (Intercept) | 58.015 | 4.767 | 12.170 | 0.000 |
| demo_raceBlack or African American | 18.102 | 8.397 | 2.156 | 0.044 |
| demo_ethnicityUnknown | -9.431 | 12.416 | -0.760 | 0.457 |
| intra_dominant_stone_size | -0.429 | 0.206 | -2.078 | 0.052 |

**Multivariable analysis on Day 14.**

**Pain Intensity**

| row_names | Estimate | Std. Error | t value | Pr(>\|t\|) |
| --- | --- | --- | --- | --- |
| (Intercept) | 42.538 | 2.380 | 17.872 | 0.000 |
| intra_preop_pcnYes | -7.871 | 4.558 | -1.727 | 0.115 |
| intra_dispo_meds___9Yes | 8.562 | 5.322 | 1.609 | 0.139 |

**Pain Interference**

| row_names | Estimate | Std. Error | t value | Pr(>\|t\|) |
| --- | --- | --- | --- | --- |
| (Intercept) | 35.778 | 6.487 | 5.515 | 0.000 |
| intra_dominant_stone_size | 0.566 | 0.272 | 2.083 | 0.064 |

**Ability to participate social**

| row_names | Estimate | Std. Error | t value | Pr(>\|t\|) |
| --- | --- | --- | --- | --- |
| (Intercept) | 51.773 | 6.098 | 8.490 | 0.000 |
| demo_raceBlack or African American | 13.627 | 11.785 | 1.156 | 0.268 |
| demo_genderWomen | -7.960 | 6.748 | -1.180 | 0.259 |
| intra_stone_locationRenal Pelvis | 8.033 | 6.461 | 1.243 | 0.236 |

**Multivariable analysis on Day 30.**

**Pain Intensity**

| row_names | Estimate | Std. Error | t value | Pr(>\|t\|) |
| --- | --- | --- | --- | --- |
| (Intercept) | 51.950 | 10.619 | 4.892 | 0.000 |
| demo_genderWomen | 4.350 | 7.685 | 0.566 | 0.582 |
| intra_stone_locationLower Pole | -4.467 | 8.461 | -0.528 | 0.607 |
| intra_stone_locationRenal Pelvis | -15.800 | 10.363 | -1.525 | 0.153 |
| intra_stone_locationUPJ | -20.700 | 8.974 | -2.307 | 0.040 |

**Pain Interference**

| row_names | Estimate | Std. Error | t value | Pr(>\|t\|) |
| --- | --- | --- | --- | --- |
| (Intercept) | 44.613 | 12.187 | 3.661 | 0.005 |
| intra_stone_locationLower Pole | -0.488 | 8.748 | -0.056 | 0.957 |
| intra_stone_locationRenal Pelvis | -6.997 | 10.096 | -0.693 | 0.506 |
| intra_stone_locationUPJ | -9.540 | 10.934 | -0.872 | 0.406 |
| demo_genderWomen | 9.788 | 6.165 | 1.588 | 0.147 |
| intra_dominant_stone_size | 0.492 | 0.236 | 2.081 | 0.067 |

**Ability to participate social**

| row_names | Estimate | Std. Error | t value | Pr(>\|t\|) |
| --- | --- | --- | --- | --- |
| (Intercept) | 54.511 | 2.989 | 18.237 | 0.000 |
| intra_insuranceMedicare | 9.129 | 5.002 | 1.825 | 0.091 |
| intra_insuranceMedicaid | -7.611 | 7.010 | -1.086 | 0.297 |
| intra_insuranceFederal/Military/Veterans Administration | -10.611 | 9.452 | -1.123 | 0.282 |

**Supplementary Table 3. Final multivariable model for severe symptoms (delta T-score >10) at post-operative day 1**

**Reduced multivariable model for the outcome: delta change in pain intensity is > 10.**

|  | Odds Ratio | 95%CI lower | 95%CI upper | p-value |
| --- | --- | --- | --- | --- |
| (Intercept) | 211003261371.217 | 0.282 | 1888683832884892156215634164983686808731648.000 | 0.310 |
| BMI | 0.442 | 0.053 | 0.952 | 0.281 |
| intra_dominant_stone_size | 0.902 | 0.550 | 1.220 | 0.595 |
| intra_same_day_dispoYes | 80.626 | 0.145 | 132877496.960 | 0.384 |

**Reduced multivariable model for the outcome: delta change in pain interference is >10.**

|  | Odds Ratio | 95%CI lower | 95%CI upper | p-value |
| --- | --- | --- | --- | --- |
| (Intercept) | 0.029 | 0 | 1.438 | 0.106 |
| intra_surgery_time | 1.029 | 1 | 1.069 | 0.082 |

**Multivariable model for the outcome: delta change in ability to participate social is < -10.**

(*The dataset is too small, unable to generate 95%CI)

|  | Odds Ratio | p-value |
| --- | --- | --- |
| (Intercept) | 0.000 | 1 |
| demo_raceBlack or African American | 2618.974 | 1 |
| intra_insuranceMedicare | 0.000 | 1 |
| intra_insuranceMedicaid | 0.000 | 1 |
| intra_insuranceFederal/Military/Veterans Administration | 0.000 | 1 |
| intra_dominant_stone_size | 0.370 | 1 |
| intra_surgery_time | 1.918 | 1 |
| intra_same_day_dispoYes | 325.484 | 1 |
| intra_dispo_meds___3Yes | 0.101 | 1 |
| intra_dispo_meds___7Yes | 710332199.325 | 1 |
