# Supplementary material for: Post‐operative recovery of quality‐of‐life following percutaneous nephrolithotomy: The impact on pain intensity and interference and the ability to participate in social roles
Source: BJUI Compass. 2025 Nov 29;6(12):e70102. doi: 10.1002/bco2.70102 (PMC12663602; doi:10.1002/bco2.70102)
Supplement: Supplementary file 3 — Data S1. Supporting Information. [file BCO2-6-e70102-s003.docx]

*Surgical Technique*

After induction of general anesthesia with placement of an endotracheal tube, flexible cystourethroscopy is performed and a 5 Fr open-ended ureteral catheter is used to cannulate the target ureter and advance into the renal pelvis. A Foley catheter is then placed for bladder drainage. Following this, patients are repositioned into the prone position. Retrograde pyelogram is performed to assess the anatomy of the collecting system and localize the stone burden. Supplemental imaging with limited renal ultrasound is also used to evaluate renal parenchyma and confirm stone location. A posterior calyx free of adjacent structures is selected for percutaneous access. Under real-time ultrasound and fluoroscopic guidance, an 18-gauge access needle is advanced using a Bullseye technique to puncture the desired calyx. After advancement of a guidewire down the ureter, the tract is sequentially dilated. A 30 Fr NephroMax balloon is then inflated to 20 atmospheres under fluoroscopic guidance and an access sheath is inserted into the collecting system. A rigid nephroscope is used to perform pyeloscopy, and stones are fragmented using an ultrasonic lithotripter. A flexible cystoscope and ureteroscope is subsequently used to evaluate the entire collecting system and ureter to confirm complete stone clearance. A nephrostomy tube is then placed into the renal pelvis and a post-placement nephrostogram is obtained to confirm appropriate positioning and unobstructed drainage. After removal of the access sheath hemostasis is achieved with direct manual compression. The nephrostomy tube is secured to the skin, and the incision is closed with absorbable suture. Patients are monitored postoperatively and typically discharged within 24 hours (median length of stay is 1 day). The nephrostomy tube is removed on post-operative day (POD) #4-6 depending on staff availability in the clinic. The first post-operative imaging with a renal ultrasound is not scheduled until approximately 2-3 months after discharge.

*Statistical Analysis*

First, a univariate analysis was performed to identify demographic (at day 0) and demographic and surgical factors (at day 1) significantly associated with a change in total T-score for each instrument at day 1. Any variable on univariate analysis with a p<0.10 was included into a multivariable linear regression model which was adjusted for insurance status (commercial, Medicare, Medicaid); stone laterality, stone location, stone size; presence of a pre-operative ureteral stent; presence of a post-operative stent. Finally, a reduced multivariable model was generated to account for categories (covariates) with very small number of participants and to reduce model overfitting due to the large number of categories. A p-value <0.05 was considered statistically significant. All analyses were performed with Stata (version 18, College Station, Texas).
