# Supplementary material for: Post‐operative recovery of quality‐of‐life following percutaneous nephrolithotomy: The impact on pain intensity and interference and the ability to participate in social roles
Source: BJUI Compass. 2025 Nov 29;6(12):e70102. doi: 10.1002/bco2.70102 (PMC12663602; doi:10.1002/bco2.70102)
Supplement: Supplementary file 3 [file BCO2-6-e70102-s002.docx]

Pain Intensity – Scale

Please respond to each item by marking one box per row.

	**In the past 7 days…**	**Had no pain**	**Mild**	**Moderate**	**Severe**	**Very severe**
PAINQU6	How intense was your pain at its worst?....	☐ 1	☐ 2	☐ 3	☐ 4	☐ 5
PAINQU8	How intense was your average pain?.........	☐ 1	☐ 2	☐ 3	☐ 4	☐ 5
		**No pain**	**Mild**	**Moderate**	**Severe**	**Very severe**
PAINQU21	What is your level of pain right now?........	☐ 1	☐ 2	☐ 3	☐ 4	☐ 5

Pain Interference – Short Form 8a

Please respond to each question or statement by marking one box per row.

	**In the past 7 days…**	**Not at all**	**A little bit**	**Somewhat**	**Quite a bit**	**Very much**
PAININ9	How much did pain interfere with your day to day activities?	☐ 1	☐ 2	☐ 3	☐ 4	☐ 5
PAININ22	How much did pain interfere with work around the home?	☐ 1	☐ 2	☐ 3	☐ 4	☐ 5
PAININ31	How much did pain interfere with your ability to participate in social activities?	☐ 1	☐ 2	☐ 3	☐ 4	☐ 5
PAININ34	How much did pain interfere with your household chores?	☐ 1	☐ 2	☐ 3	☐ 4	☐ 5
PAININ12	How much did pain interfere with the things you usually do for fun?	☐ 1	☐ 2	☐ 3	☐ 4	☐ 5
PAININ36	How much did pain interfere with your enjoyment of social activities?	☐ 1	☐ 2	☐ 3	☐ 4	☐ 5
PAININ3	How much did pain interfere with your enjoyment of life?	☐ 1	☐ 2	☐ 3	☐ 4	☐ 5
PAININ13	How much did pain interfere with your family life?	☐ 1	☐ 2	☐ 3	☐ 4	☐ 5

Ability to Participate in Social Roles and Activities – Short Form 8a

Please respond to each item by marking one box per row.

		Never	Rarely	Sometimes	Usually	Always
SRPPER11_ CaPS	I have trouble doing all of my regular leisure activities with others	☐ 5	☐ 4	☐ 3	☐ 2	☐ 1
SRPPER18_ CaPS	I have trouble doing all of the family activities that I want to do......................	☐ 5	☐ 4	☐ 3	☐ 2	☐ 1
SRPPER23_ CaPS	I have trouble doing all of my usual work (include work at home)...........................	☐ 5	☐ 4	☐ 3	☐ 2	☐ 1
SRPPER46_ CaPS	I have trouble doing all of the activities with friends that I want to do...................	☐ 5	☐ 4	☐ 3	☐ 2	☐ 1
SRPPER15_ CaPS	I have to limit the things I do for fun with others...	☐ 5	☐ 4	☐ 3	☐ 2	☐ 1
SRPPER28r1	I have to limit my regular activities with friends	☐ 5	☐ 4	☐ 3	☐ 2	☐ 1
SRPPER14r1	I have to limit my regular family activities..	☐ 5	☐ 4	☐ 3	☐ 2	☐ 1
SRPPER26_ CaPS	I have trouble doing all of the work that is really important to me (include work at home)	☐ 5	☐ 4	☐ 3	☐ 2	☐ 1
